# Supplementary material for: Genetic and Structural Diversity of Prokaryotic Ice-Binding Proteins from the Central Arctic Ocean
Source: Genes (Basel). 2023 Jan 30;14(2):363. doi: 10.3390/genes14020363 (PMC9957290; doi:10.3390/genes14020363)
Supplement: Supplementary file 1 [file genes-14-00363-s001.zip › genes-2129580-supplementary.pdf]

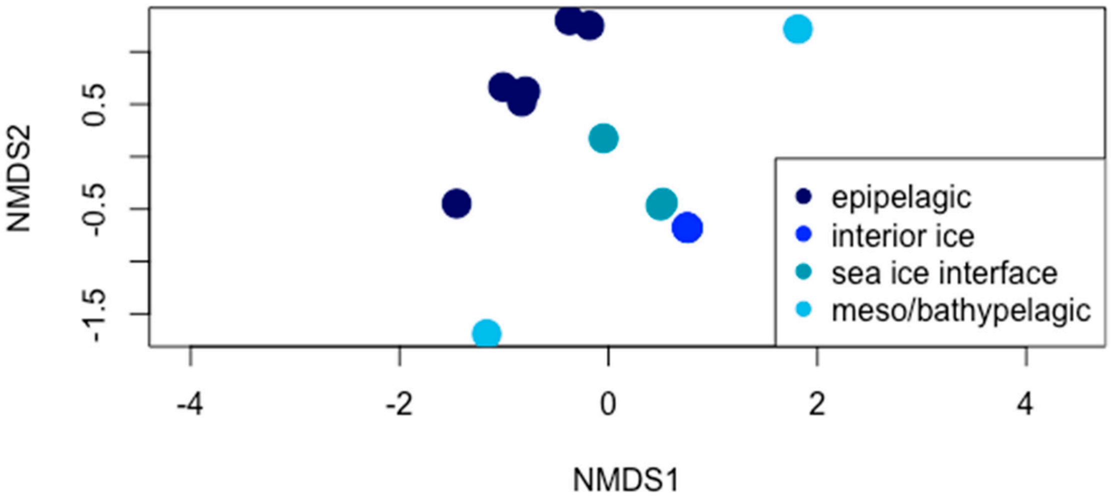

Supplementary Figure S1: Non-metric multidimensional scaling of order-level community composition across different environments. Note that interior ice has 4 data points overlapping in the same position.

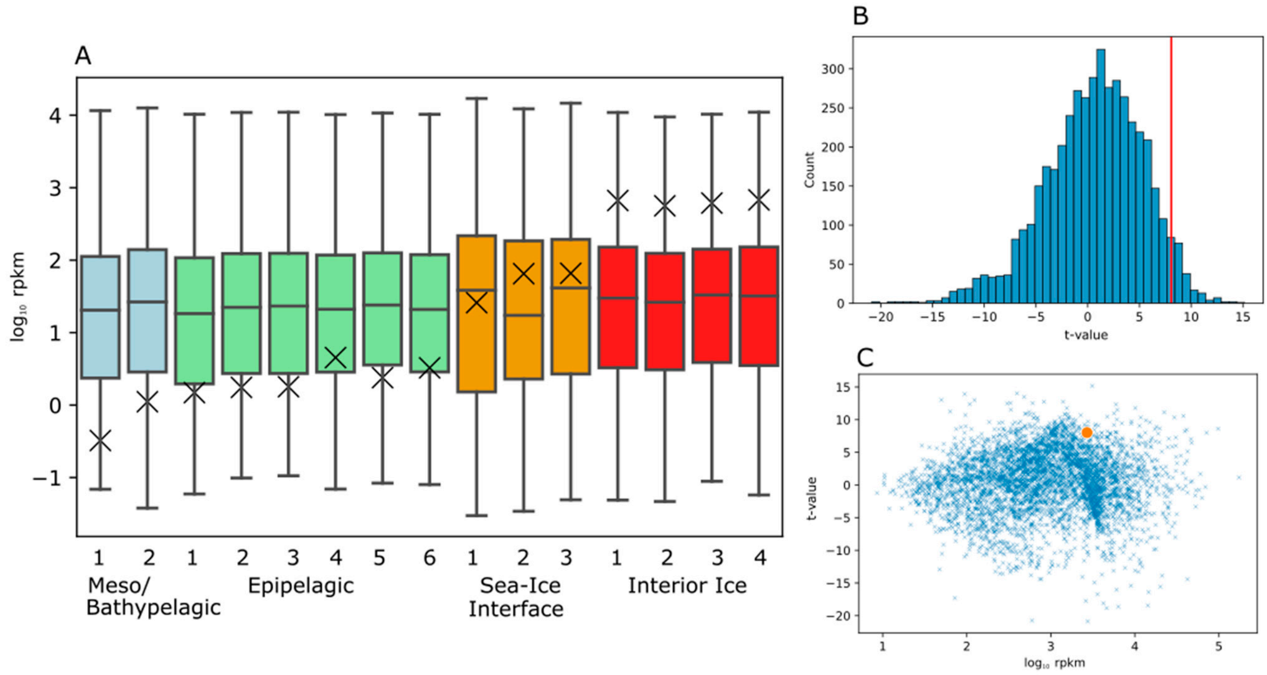

Supplementary Figure S2: PF11999 (DUF3494) domains are one of the most differentially abundant Pfams when comparing ice and water.

Panel A: Boxplots of Pfam abundances within the prokaryotic community (log RPKM) across samples; the crosses signify total abundance of the DUF3494 domain. Panel B: Histogram showing t-values of all Pfams present at a moderate level (total RPKM > 50), when comparing ice to water (Welch's t-test, log RPKM). The red vertical line (8.01) is the t-value of the DUF3494 domain. Panel C: scatterplot showing t-

values and abundance (log RPKM) of all 8012 Pfams present across the samples. The orange dot indicates the DUF3494 domain. Most Pfams are less differentially abundant when comparing ice to water (lower t-value), or otherwise less abundant overall (lower RPKM).

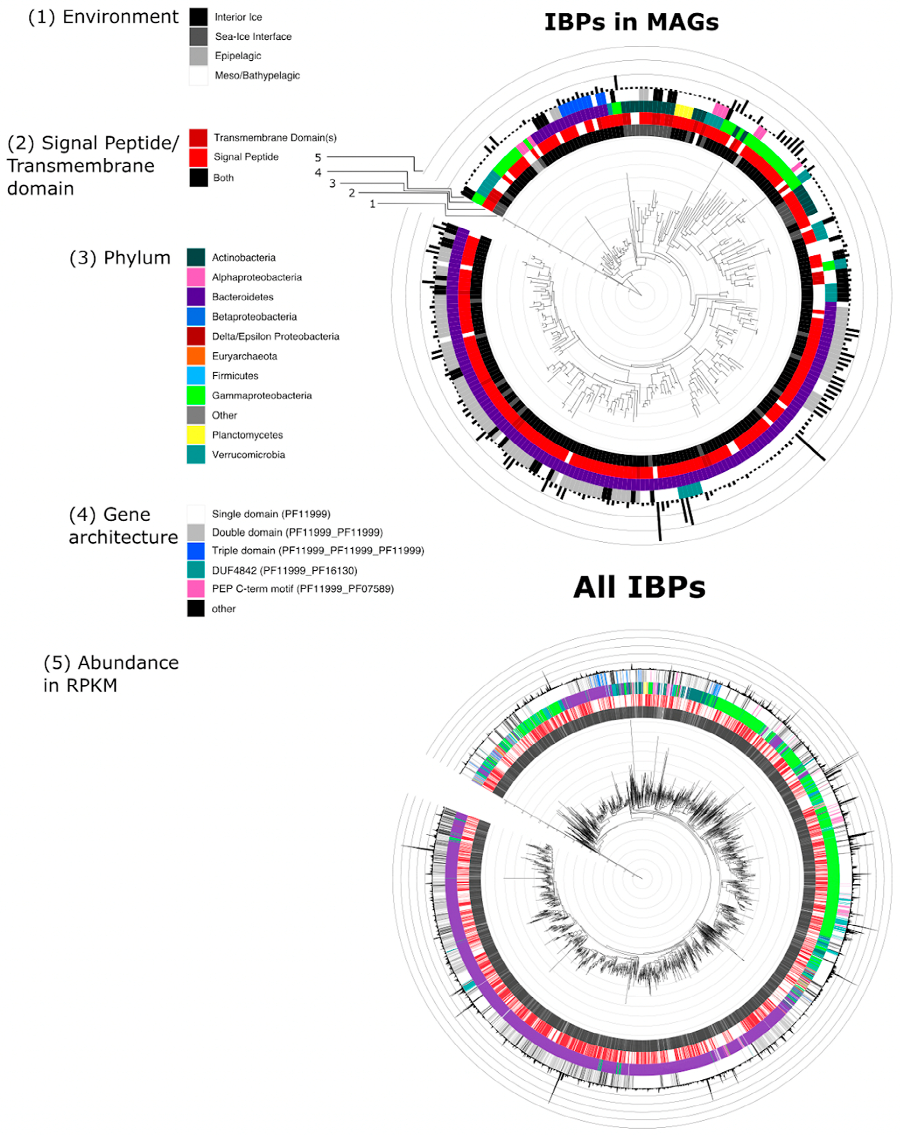

Supplementary Figure S3: Trees of IBPs from MAGs and total assembly.

Supplementary Table S1: Sample location, processing and sequencing data.

Label	Sample Collection Date	Depth (m)*	Habitat	Latitude	Longitude	Pooled?	Illumina Regular or Low Input 300 bp protocol	Filter Volume (mL)
sea_ice_interface_1	13/01/2020	0.00- 0.05	Sea Ice	87.3239	107.4423		Low input	1220
epipelagic_1	16/01/2020	51	Seawater	87.5518	102.0858		regular	8500
epipelagic_2	16/01/2020	51	Seawater	87.5518	102.0858		regular	8500
epipelagic_3	16/01/2020	51	Seawater	87.5518	102.0858	Yes, from epipelagic 1 and 2	regular	n/a
sea_ice_interface_2	27/01/2020	0.00 - 0.05	Sea Ice	87.4458	95.6702		Low input	1230
interior_ice_1	03/02/2020	0.30 - 0.40	Sea Ice	87.4122	93.2151		regular	2920
interior_ice_2	03/02/2020	0.05 - 0.30	Sea Ice	87.4122	93.2151		regular	2150
interior_ice_3	03/02/2020	0.50 - 0.60	Sea Ice	87.4122	93.2151		regular	1930
interior_ice_4	03/02/2020	0.40 - 0.50	Sea Ice	87.4122	93.2151		regular	1180
sea_ice_interface_3	03/02/2020	0.00 - 0.05	Sea Ice	87.4122	93.2151		Low input	1500
epipelagic_4	06/02/2020	20	Seawater	87.5953	94.0846	Yes, from epipelagic 5 and 6	regular	n/a
epipelagic_5	06/02/2020	20	Seawater	87.5953	94.0846		regular	5500
epipelagic_6	06/02/2020	20	Seawater	87.5953	94.0846		regular	8500
meso/bathypelagic_1	06/02/2020	202	Seawater	87.5953	94.0846		Low input	9500
meso/bathypelagic_2	07/02/2020	4082	Seawater	87.6362	93.7496		Low input	6000

*For sea-ice samples, depth is relative to the sea-ice interface.

Supplementary Table S2: Sample IDs (Label used in this paper, MOSAiC, GOLD, JGI, and IMG/M, IDs). Data can be accessed through the JGI IMG/M web portal under proposal: The International Arctic Ice Drift Experiment MOSAiC: Seasonal changes of microbial communities across the Arctic Ocean (Proposal ID: 505419)

Label	MOSAiC Sample Identifier	GOLD ID	JGI ID	IMG/M ID
sea_ice_interface_1	PS122_totDNA_309	Gp0561256	1290821	3300045789

epipelagic_1	PS122_totDNA_328	Gp0561257	1290823	3300046532
epipelagic_2	PS122_totDNA_327	Gp0561266	1292144	3300046450
epipelagic_3	PS122_totDNA_327_328_pool	Gp0561269	1292150	3300047669
sea_ice_interface_2	PS122_totDNA_371	Gp0561258	1290827	3300045790
interior_ice_1	PS122_totDNA_414	Gp0561263	1290837	3300049783
interior_ice_2	PS122_totDNA_411	Gp0561260	1290831	3300046534
interior_ice_3	PS122_totDNA_412	Gp0561261	1290833	3300047666
interior_ice_4	PS122_totDNA_413	Gp0561262	1290835	3300047667
sea_ice_interface_3	PS122_totDNA_405	Gp0561259	1290829	3300046449
epipelagic_4	PS122_totDNA_424_425_pool	Gp0561270	1292152	3300046103
epipelagic_5	PS122_totDNA_424	Gp0561267	1292146	3300046451
epipelagic_6	PS122_totDNA_425	Gp0561268	1292148	3300047668
meso/bathypelagic_1	PS122_totDNA_418	Gp0561264	1290839	3300046467
meso/bathypelagic_2	PS122_totDNA_432	Gp0561265	1290841	3300045738

Supplementary Table S3: Assembly statistics

Label	Raw base count	Raw read count	Filtered base count	Filtered read count	Number of contigs	Contig N50 (bases)	Contig N90 (bases)	Reads aligned to assembly	Reads aligned to assembly (%)
sea_ice_interface_1	84577345974	560114874	8.3411E+10	556639888	1263564	941	314	526918831	94.7
epipelagic_1	66382153108	439616908	6.4723E+10	433300950	3535797	961	364	265263982	61.2
epipelagic_2	46649574580	308937580	4.393E+10	295072406	2727462	883	306	170433025	57.8
epipelagic_3	46925855958	310767258	4.1761E+10	280536486	2461643	915	306	159271165	56.8
sea_ice_interface_2	78632944074	520747974	7.6254E+10	509156444	2267533	779	302	457576800	89.9
interior_ice_1	53757462586	356009686	5.3096E+10	354779856	2152549	1227	327	304698615	85.9
interior_ice_2	64519501366	427281466	6.3638E+10	425000974	2416493	1605	362	372454027	87.6
interior_ice_3	38998755202	258269902	3.8395E+10	256465328	1538500	1442	347	213781315	83.4
interior_ice_4	52344870874	346654774	5.158E+10	344450412	2045557	1208	332	295470615	85.8
sea_ice_interface_3	56780937398	376032698	5.4565E+10	364388456	1661614	707	304	325070478	89.2
epipelagic_4	628610	4162985	6.0507E	4064715	3482628	784	300	26359788	64.9

	75010	10	+10	84				8	
epipelagic_5	440197 71264	2915216 64	4.1953E +10	2817164 88	2461877	853	306	17867599 3	63.4
epipelagic_6	514677 41470	3408459 70	5.001E+ 10	3354278 02	3050031	773	296	21505034 8	64.1
meso/bathypelagic_1	569478 91152	3771383 52	5.586E+ 10	3742934 82	3822139	766	316	25559932 9	68.3
meso/bathypelagic_2	818958 59284	5423566 84	8.0629E +10	5390569 76	3094213	1067	311	46445600 2	86.2

Supplementary Table S4: List of *Polarella glacialis* IBP accessions from the NCBI Short Read Archive (SRA), BioProject accession PRJEB33539

CAE8729154.1	CAE8679677.1	CAE8631646.1	CAE8583686.1	CAE8583687.1	CAE8636840.1	CAE8636836.1	CAE8582104.1	CAE8583689.1
CAE8636834.1	CAE8583688.1	CAE8582102.1	CAE8582101.1	CAE8631645.1	CAE8582103.1	CAE8631647.1	CAE8679681.1	CAE8729160.1
CAE8729152.1	CAE8618267.1	CAE8677481.1	CAE8741406.1	CAE8583685.1	CAE8637704.1	CAE8618265.1	CAE8639353.1	CAE8634595.1
CAE8639355.1	CAE8637703.1	CAE8729150.1	CAE8582100.1	CAE8581014.1	CAE8741407.1	CAE8618268.1	CAE8639358.1	CAE8636643.1
CAE8688622.1	CAE8639354.1	CAE8639356.1	CAE8636651.1	CAE8585100.1	CAE8628465.1	CAE8588114.1	CAE8595931.1	CAE8636649.1
CAE8636648.1	CAE8671768.1	CAE8671762.1	CAE8671764.1	CAE8636642.1	CAE8671759.1	CAE8636644.1	CAE8640504.1	CAE8696859.1
CAE8625316.1	CAE8634924.1	CAE8636647.1	CAE8604292.1	CAE8607075.1	CAE8668303.1	CAE8654806.1	CAE8643832.1	CAE8652165.1
CAE8601668.1	CAE8671765.1	CAE8636645.1	CAE8720042.1	CAE8717284.1	CAE8611419.1	CAE8622661.1	CAE8618266.1	CAE8677484.1
CAE8640451.1	CAE8647134.1	CAE8635871.1	CAE8652167.1	CAE8622663.1	CAE8581017.1	CAE8635872.1	CAE8594356.1	CAE8643833.1
CAE8594357.1	CAE8647038.1	CAE8641817.1	CAE8647135.1	CAE8588179.1	CAE8585364.1	CAE8654565.1	CAE8650135.1	CAE8602834.1
CAE8717287.1	CAE8743901.1	CAE8706293.1	CAE8729157.1	CAE8591189.1	CAE8629564.1	CAE8649369.1	CAE8675450.1	CAE8634922.1
CAE8700145.1	CAE8613500.1	CAE8623192.1	CAE8647136.1	CAE8616734.1	CAE8631824.1	CAE8588201.1	CAE8627979.1	CAE8720727.1
CAE8688779.1	CAE8616733.1	CAE8607076.1	CAE8706292.1	CAE8616732.1	CAE8687224.1	CAE8624346.1	CAE8675449.1	CAE8625315.1
CAE8743391.1	CAE8723718.1	CAE8624936.1	CAE8642055.1	CAE8720725.1	CAE8743390.1	CAE8702088.1	CAE8588202.1	CAE8613502.1
CAE8588206.1	CAE8588203.1	CAE8703796.1	CAE8671446.1	CAE8690017.1	CAE8681934.1	CAE8613501.1	CAE8598188.1	CAE8687064.1

Supplementary Table S5: Genomic context of IBPs from selected MAGs.

Organism	architecture	contig ID	IBP position	Upstream Pfams	Upstream position	Upstream grouping	Downstream Pfams	Downstream position	Downstream grouping
Crocinitomicaceae; UBA4466	11999_11999	Ga0491196_0000314	41016_44504	pfam13412	39626 - 40375	DNA_binding	pfam13532	45866_46459	oxygenase
	11999_11999	Ga0491196_0000991	29281_31188	pfam00132	26291_26482	hexapeptide_repeat	pfam13585_pfam0080	31280_33334	secretion_ Ig_containing
	11999_11999	Ga0491196_0002878	3075_5033	NA			pfam01609	5440_5775	transposase
	11999_11999	Ga0491196_0004603	1035_4541	pfam01527 (	589_903	transposase	pfam13369	6504_7412	transglutaminase
	11999_11999	Ga0491196_0006284_	5019_6899	pfam11251	4103_4885	redox-related	pfam01527	7633_7914	transposase
	11999_11999	Ga0491196_0006863	7168_9048	pfam04185	5000_7027	phosphoesterase	pfam05970	10165_12465	helicase
Flavobacteriaceae; GCA-002733185	pfam11999_pfam07603	Ga0491197_0011800	1860_3128	pfam02110	88_879	kinase	pfam12740	4839_5759	chlorophyllase
	pfam11999_pfam11999	Ga0491197_0008641	2_1189	NA			pfam00009	1591_1962	translation (elongation factor TU)
	pfam11999	Ga0491197_0005030	61_1332	NA			pfam11999	1406_2662	IBP
	pfam11999	Ga0491197_0005030	2831_4087	pfam11999	1406_2662	IBP	pfam00664_pfam00005	4853_6589	transporter_transporter (ABC transporter)
	pfam11999	Ga0491197_0005030	1406_2662	pfam11999	61_1332	IBP	pfam11999	2831_4087	IBP

	pfam11999	Ga0491197_0004111	3_929	NA			pfam00501	2273_3289	AMP-binding
	pfam11999	Ga0491197_0001959	1113_1889			IBP	pfam08695	3738_4082	cytochrome_oxidase
	pfam11999	Ga0491197_0001959	1_390	NA			pfam11999	1113_1889	IBP
	pfam11999_pfam11999_pfam01345	Ga0491197_0001071	33995_36562	pfam00009_pfam16658	32025_33611	translation (elongation factor TU)_translation (translation release factor)	NA		
Myxococcota; UBA796; GCA-2862545	pfam11999	Ga0491196_0003486	10900_11541	pfam11617 x 10	5868_8915	metal_binding_motif	pfam03050	13929_14261	transposase
	pfam11617_pfam11999	Ga0491196_0003486	14591_16588	pfam03050	13929_14261	transposase	pfam04389	16901_17839	peptidase
	pfam02412_pfam11999	Ga0491196_0012452	874_2703	NA			pfam01380_pfam01380_pfam13537	6209_7717	sugar isomerase & amidotransferase
Spirosomaceae; Unknown	pfam11999_pfam11999_pfam11999	Ga0491197_0000463	45380_47548	pfam14684_pfam14685_pfam03572_pfam07676 (x5)	41907_45206	protease_peptidase_cell_surface protein	pfam03313	48074_49609	Serine dehydratase
Bacteroidia; NS11-12g; UBA9320	pfam11999_pfam11999	Ga0491197_0005799	3232_5151			IBP			IBP
	pfam11999_pfam11999	Ga0491197_0005799	5881_7797			IBP			IBP

	pfam11999_ pfam11999	Ga0491197_0005799	782_2698	NA	NA	NA			IBP
	pfam11999_ pfam11999	Ga0491197_0005799	8860_10776			IBP	pfam04055	11264_12343	SAM superfamily
